# Venous Leak Embolization in Patients with Venogenic Erectile Dysfunction via Deep Dorsal Penile Vein Access: Safety and Early Efficacy

**DOI:** 10.1007/s00270-023-03412-2

**Published:** 2023-03-22

**Authors:** N. Diehm, S. Pelz, C. Kalka, H. H. Keo, V. Mohan, M. C. Schumacher, D. D. Do, H. Hoppe

**Affiliations:** 1 Vascular Institute Central Switzerland, Aarau, Switzerland; 2 University of Applied Sciences Furtwangen, Villingen-Schwenningen, Germany; 3 Department of Angiology, University Hospital of Basel, Basel, Switzerland; 4 University of Bern, Bern, Switzerland; 5 Department of Urology, Hirslanden Clinic Aarau, Aarau, Switzerland; 6 SwissIntervention Microtherapy Center, Bern, Switzerland; 7 Campus Stiftung Lindenhof Bern, Swiss Institute for Translational and Entrepreneurial Medicine, Bern, Switzerland; 8 University of Lucerne, Lucerne, Switzerland

**Keywords:** Erectile dysfunction, Embolization, Venous incompetence, Venous leak, Corpus cavernosum

## Abstract

**Purpose:**

This all-comers registry aimed to assess safety and early efficacy of venous embolization in patients with venogenic erectile dysfunction due to venous leak in an unselected cohort.

**Methods:**

Between October 2019 and September 2022, patients with venogenic erectile dysfunction resistant to phosphodiesterase-5-inhibitors were treated with venous embolization using ultrasound-guided anterograde access via a deep dorsal penile vein in a single center. A mix of ethiodized oil and modified cyanoacrylate-based glue n-butyl 2 cyanoacrylate (NBCA) monomer plus methacryloxy-sulpholane monomer (Glubran-2, GEM, Italy) was used as liquid embolic agent. Prior to embolization, venous leak had been verified based on penile duplex sonography and computed tomography cavernosography. Procedural success was defined as technically successful and complete target vein embolization. The primary safety outcome measure was any major adverse event 6 weeks after the procedure. The primary feasibility outcome measure was IIEF-15 (International Index of Erectile Function-15) score improvement ≥ 4 points in ≥ 50% of subjects on 6 weeks follow-up post intervention.

**Results:**

Fifty consecutive patients (mean age 61.8 ± 10.0 years) with severe erectile dysfunction due to venous leak underwent venous embolization. Procedural success was achieved in 49/50 (98%) of patients with no major adverse events on follow-up. The primary feasibility outcome measure at 6 weeks was reached by 34/50 (68%) of patients.

**Conclusion:**

Venous leak embolization via deep dorsal penile vein access using a liquid embolic agent was safe for all and efficacious in the majority of patients with severe venogenic erectile dysfunction on 6 weeks follow-up.

**Supplementary Information:**

The online version contains supplementary material available at 10.1007/s00270-023-03412-2.

## Introduction

Erectile dysfunction (ED) is a common disease with a reported overall prevalence of 18–49% in men, increasing with age and cardiovascular risk factors. The worldwide prevalence was predicted to increase from 152 million cases in 1995–322 million by the year 2025 [1].

The main causes of organic ED are vascular, endocrine, or drug related. Inability to achieve or maintain an erection for satisfactory sexual intercourse has a significant impact on patients’ quality of life, self-confidence, and interpersonal relations.

During the past two decades, treatment of ED has evolved considerably and was revolutionized by the introduction of Phosphodiesterase-5 inhibitors (PDE5i), which are considered the first-line therapy for ED. However, up to 50% of patients on PDE5i report on insufficient erections for intercourse or have relevant side effects [2].

It has been previously reported that ED patients who are non-responsive to PDE5i have a high likelihood of vascular etiology [3]. Venous leak and arterial obstruction each constitute about 50% of vascular causes leading to ED symptoms refractory to vasoactive agents.

Venogenic ED occurs when incomplete relaxation of the cavernosal smooth muscle during arterial inflow fails to occlude penile venous outflow tracts. The etiology of venogenic ED is not yet fully understood. It was previously reported that venogenic ED may relate to age or injury-related changes in the tunica albuginea, cavernosal smooth muscle dysfunction due to structural alterations, excessive adrenergic input or from shunts that may have developed during previous priapism episodes and subsequent repair [4, 5]. Potential risk factors for the development of veno-occlusive dysfunction may be age, diabetes, prostatectomy, pelvic radiation, and androgen deprivation therapy [6].

Previously, venous leak was treated using various approaches including surgical ligation and percutaneous embolization utilizing either a retrograde transvenous or anterograde via deep dorsal penile veins with or without surgical cut down [7]. Surgical treatment is rather invasive and usually has to be performed in an operating room under general anesthesia. In addition, long-term success rates of surgical ligation of the deep dorsal vein and its collaterals were reported to be approximately 25% [8, 9]. The aim of endovascular therapy is sufficient embolization of efferent pelvic veins such as periprostatic, internal or external pudendal veins. Venous embolization via a deep dorsal penile vein was previously reported as endovascular treatment method [6, 10–12].

The objective of the present study was to assess safety and early efficacy of venous leak embolization using an anterograde access via a deep dorsal penile vein in a larger consecutive ED patient population non-responsive to PDE5i.

## Patients and Methods

Patients included in the present study from October 2019 to September 2022 were from a prospective, single-center, all-comers, investigator-initiated registry based on data from patients with ED due to venous leak of one or more erection-related efferent veins and unsatisfactory response or adverse side effects to PDE5i medication or contraindications for their use. Data were collected and subsequently anonymized through a routine quality management information system.

Prior to vascular workup, all patients were investigated by a board-certified urologist. In addition, all patients underwent ample laboratory testing including renal function, cholesterol and testosterone levels. Venous leak was diagnosed and quantified by penile duplex ultrasound and contrast-enhanced computed tomography cavernosography. Key exclusion criteria for endovascular treatment according to site’s standard of care were non-vascular causes of ED including penile anatomic defects, spinal cord injury, and primary psychogenic disorders.

*Penile duplex sonography (ref. online supplement)*

*Computed tomography cavernosography (ref. online supplement)*

### Interventional Procedures

Venous leak embolization was performed after intra-cavernosal injection of 20 μg alprostadil. The patient was draped in a supine position. Following local subcutaneous administration of lidocaine 2% for local anesthesia of the proximal penis and light sedation with midazolam, an ultrasound-guide puncture of a penile deep dorsal vein was performed using a stiff 20-G micropuncture set with a 0.018-inch guide wire and a 4-French introducer with a stiff 3-F inner dilator (Cook Inc., Bloomington, Indiana, U.S.A.). A stiff 3-F inner dilator was introduced through the penile fascia (Buck’s fascia) into a deep dorsal penile vein. The introducer was carefully advanced and positioned in close proximity to the radix penis, and a diagnostic venogram was acquired confirming venous leakage. Subsequently, all materials were flushed using 5% glucose solution. Subsequently venous embolization was performed with a slow but steady injection using a liquid embolic agent modified cyanoacrylate based glue n-butyl 2 cyanoacrylate (NBCA) monomer plus methacryloxy-sulpholane monomer (Glubran-2, GEM, Italy) and ethiodized oil (Lipiodol by Guerbet, Zurich, Switzerland) mixed in 1:1–1:3 ratios under Valsalva maneuver and continuous fluoroscopic monitoring. The injection was terminated in time prior to inadvertent distribution of embolization material from internal pudendal or periprostatic veins to the iliohypogastric veins, external pudendal veins to femoral veins, or dorsal penile veins. The total amount of the liquid embolic agent was 1 to 3 ml.

In veins with fast outflow on venogram protective distal coil embolization was performed prior to embolization with liquid glue in order to prevent its distribution to femoral or iliohypogastric veins. Therefore, fibered-microcoils (Nester, Cook Inc., Bloomington, Indiana, U.S.A.) were used for transcatheter embolization with a 2.7-F microcatheter (Progreat, Terumo, Japan). Coils were oversized by 30% in relation to target vein diameter in order to prevent coil migration.

Alternatively, in case of failure of transpenile venous access, transcatheter embolization was performed using a transfemoral venous approach via internal iliac and internal pudendal veins with a liquid embolic agent as previously reported [13].

### Medical Therapy

Post-procedurally, patients were administered antibiotic medication ciprofloxacin 500 mg per os twice daily for 7 days, antiphlogistic medication ibuprofen 600 mg per os up to three times daily for 7 days, and pantoprazole 40 mg per os once daily for stomach protection. Additional oral pain medication was prescribed if necessary. Furthermore, patients were recommended tadalafil 5 mg per os once daily for 6 weeks starting 5 days after transcatheter embolization procedure.

### Outcome Assessment

To quantify erectile function before and after transcatheter embolization, all patients answered the International Index of Erectile Function-15 (IIEF-15) questionnaire, consisting of 15 standardized questions divided into the topics erectile function, orgasmic function, sexual desire, and sexual satisfaction [14–17]. The possible score per question is zero to five per question yielding a maximum of a 75 point score.

Patients received a baseline questionnaire at first presentation and follow-up-questionnaires with the same questions 6 weeks post intervention. An improvement by 4 points in the erectile function domain consisting of 6 questions (IIEF-6) was defined as minimal clinically important difference (MCID) and is considered clinically relevant. Procedural success was defined as technically successful and complete target vein embolization.

The visual analog scale (VAS, range 1–10) was used to quantify pain after the application of the embolic agent. The primary safety outcome measure was absence of device- or procedure-related death or major adverse events (MAE), such as gangrene or necrosis in the embolization area of the pelvic veins and symptomatic deep venous thrombosis or symptomatic pulmonary embolism. Complications were assessed according to the CIRSE classification [18]. The primary feasibility outcome measure was a minimally clinically relevant improvement of ≥ 4 in the IIEF-6 score at 6 weeks. The feasibility of the treatment was demonstrated when at least 50% of the patients showed an MCID [19]. In addition, responses to IIEF-15 question 3 on ability to achieve penetration, and on IIEF-15 question 4 on ability to maintain erection sufficient for sexual intercourse, considered as key components of erectile function, as well as the IIEF-15 total score were separately evaluated. Moreover, patients answered the patient global impression of improvement (PGI-I) questionnaire six weeks post intervention [20]. Improvement after embolization was evaluated with a single question to assess global treatment satisfaction: (1) very much better, (2) much better, (3) a little better, (4) no change, (5) a little worse, (6) much worse, (7) very much worse. Finally, patients were asked at 6 weeks as to whether they would undergo the procedure again.

### Statistical Design and Analysis

Continuous variables are reported as mean ± SD and categorical variables as counts and percent. Differences between means of continuous variables were assessed with Students *t* test, Mann-Whiney *U* test or Wilcoxon signed-rank test were appropriate. A two-sided value of *p* < 0.05 indicated statistical significance. Statistical analyses were performed with XLSTAT software, version 2015.6.01.24026 (Addinsoft SARL).

## Results

Fifty consecutive patients with venogenic ED were included in this study. Patient baseline demographics and comorbidities are demonstrated in Table 1. Patient baseline response to conservative therapy (PDE5-i and intracavernous alprostadil) is summarized in Table 2. Patient baseline characteristics related to ED (IIEF-15 score and findings on penile duplex sonography) are demonstrated in Table 3.

**Table 1:** Baseline Patient Demographics and Comorbidities of 50 ED patients

Age, years	61.8 ± 10.0
Cigarette smoking	13 (26)
Diabetes mellitus	6 (12)
Arterial hypertension	18 (36)
Hyperlipidaemia	28 (56)
Coronary artery disease	6 (12)
Peripheral artery disease	1 (2)
Cerebrovascular disease	0 (0)
History of arterial revascularization for ED	6 (12)
Neurological disease	2 (4)
Renal insufficiency	0 (0)
History of, or current dialysis	0 (0)
History of prostate surgery	4 (8)
History of depression	3 (6)
Chronic prostatitis	1 (2)
Hypogonadism	1 (2)
Alcoholism	2 (4)
Drug abuse	3 (6)
Consumption of drugs with impact on ED	17 (34)

ED = erectile dysfunction

Values are mean ± SD or n (%)

**Table 2:** Baseline Patient Response to Conservative Therapy (PDE5-i and intracavernous alprostadil)

No response to PDE5-I	47 (94)
Severe side effects of PDE5-I	3 (60)
No response to alprostadil	45 (90)

Values are n (%)

**Table 3:** Baseline characteristics related erectile dysfunction

Baseline IIEF-15 score	31.1	± 15.1
Baseline IIEF-3 score	1.7	± 1.6
Baseline IIEF-4 score	1.3	± 1.2
PSV left, cm/sec (*n* = 95)	34.2	± 13.4
PSV right, cm/sec (*n* = 95)	37.1	± 13.6
EDV left, cm/sec (*n* = 94)	9.7	± 5.0
EDV right, cm/sec (*n* = 95)	10.5	± 6.0

EDV, end-diastolic velocity; PSV, peak systolic velocity; IIEF-15, 15-item International Index for Erectile Dysfunction

Values are mean ± SD or n (%)

**Fig. 1:**
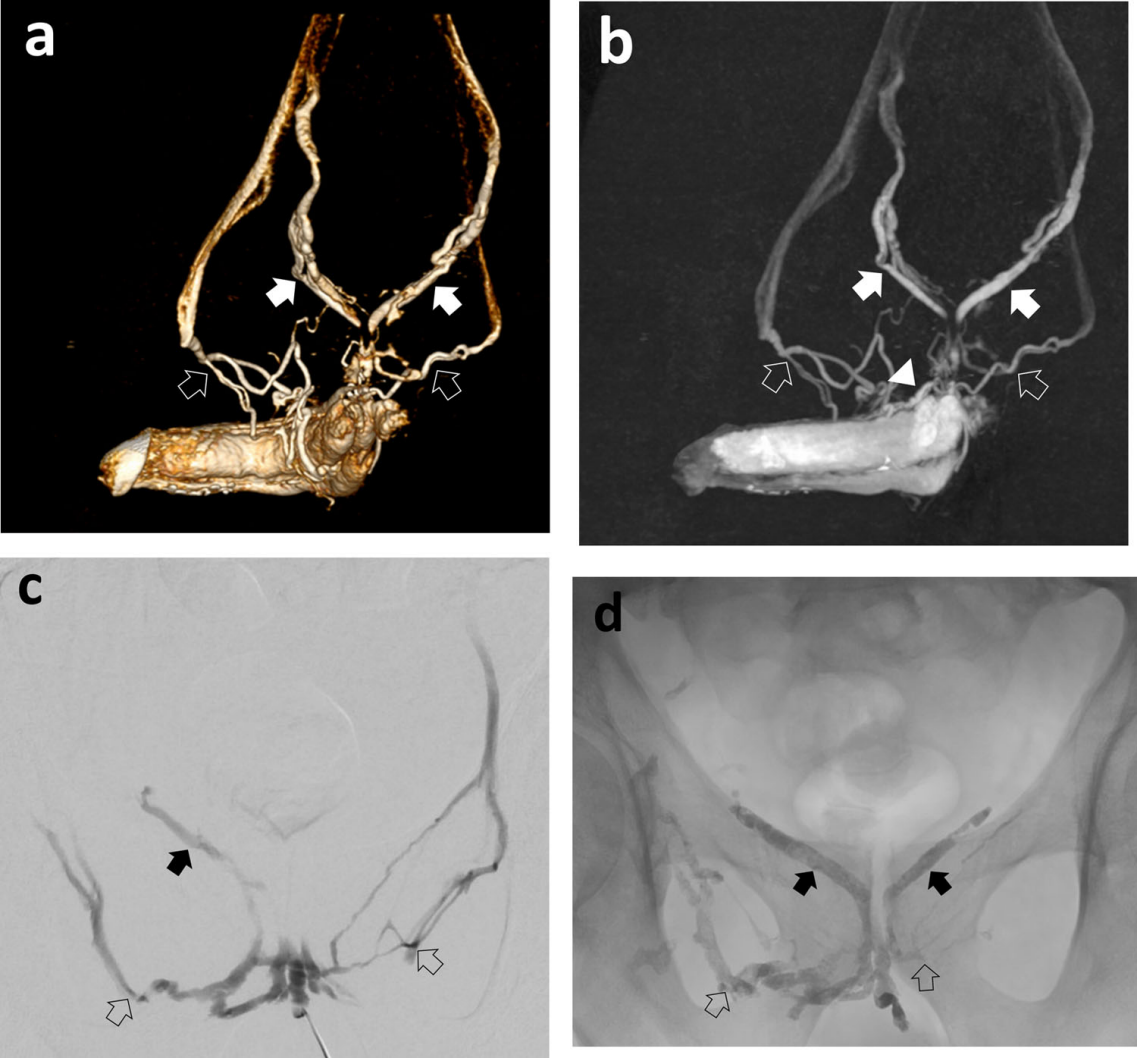
58-year-old patient with erectile dysfunction due to venous leak. Venographic depiction of a venous leak of internal (arrows) and external (open arrows) pudendal veins and large-caliber deep dorsal penile vein (arrowhead) on **a** CT cavernosography (volume rendering), **b** CT cavernosography (maximum intensity projection), **c** invasive venogram post direct puncture of the deep dorsal vein followed by embolization of large efferent veins using Glubran-2 **d**. His total IIEF-15 score improved from 20 at baseline to 67 at 6 weeks

**Fig. 2:**
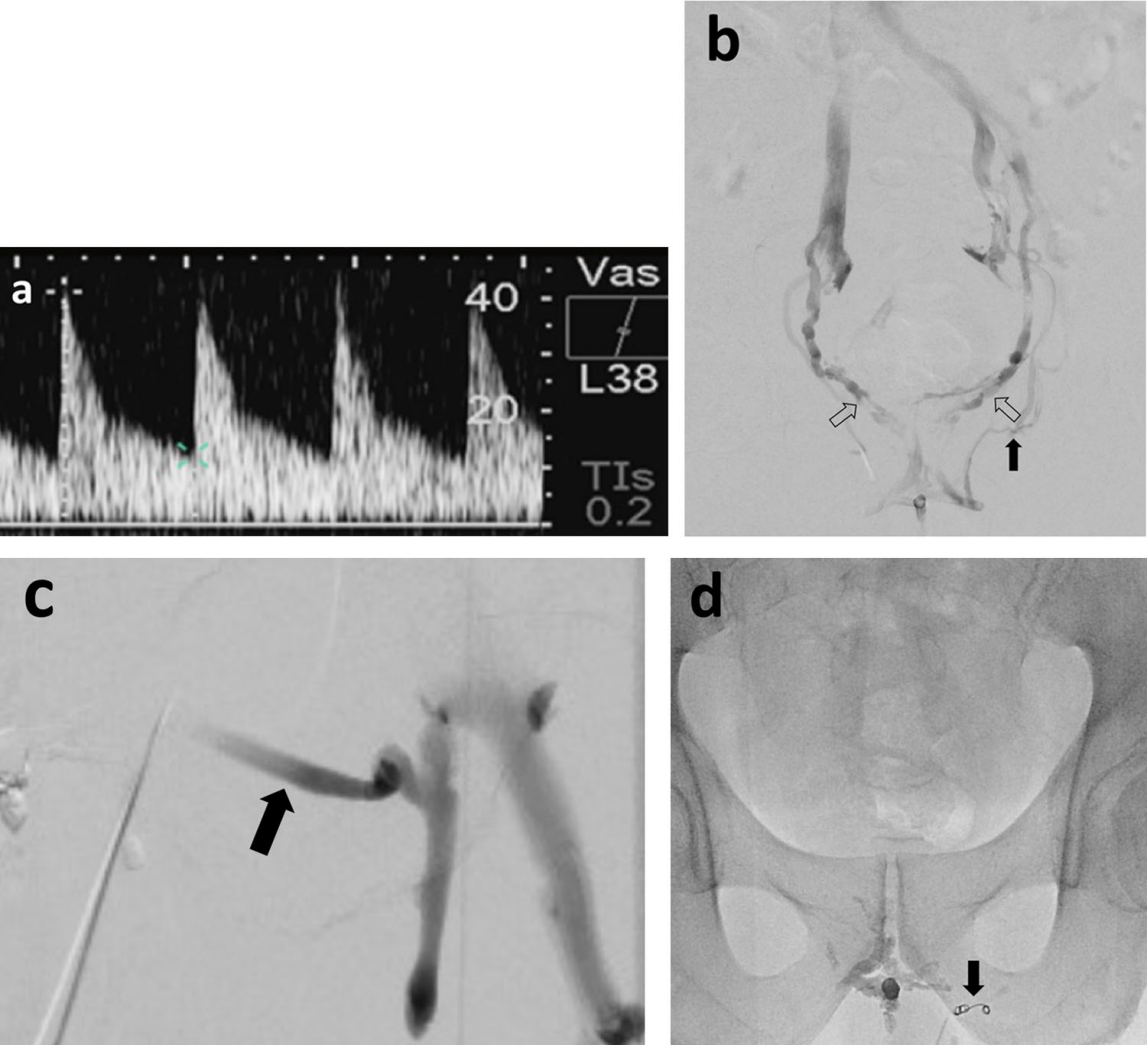
72-year-old patient with severe erectile dysfunction not responding to PDE-5-inhibitors. Penile duplex sonography demonstrated increased end-diastolic flow velocities **a**. Venogram **b** demonstrating venous leak of internal (open arrows) and external (arrow) pudendal veins with left external pudendal vein (arrow) draining in the left saphenous vein **c**. Embolization was performed using Glubran-2 after protective coil embolization of the left external pudendal vein with coils **d**

Procedural success was achieved in 49/50 (98%) of patients with no major adverse events (MAE) at follow-up (Fig. 1). In one patient, intravenous access could not be obtained due to hypoplastic deep dorsal penile veins. However, this patient eventually was treated successfully in a secondary transcatheter embolization of venous leak using a transfemoral approach. Three patients underwent protective distal coil embolization prior to embolization with liquid embolic agent in order to prevent its distribution to femoral or iliohypogastric veins (Fig. 2).

A total of 25/50 (50%) patients experienced mild to moderate pain after application of the embolic agent (mean VAS 3.86 ± 3.49). No patient experienced a MAE during the course of the study. Asymptomatic pulmonary embolisms by small amounts of the liquid embolic agents were noted by fluoroscopy in 2/50 (4%) patients. This was treated medically with 4 weeks of 10 mg of Rivaroxaban daily (CIRSE grade 3 complication).

**Fig. 3:**
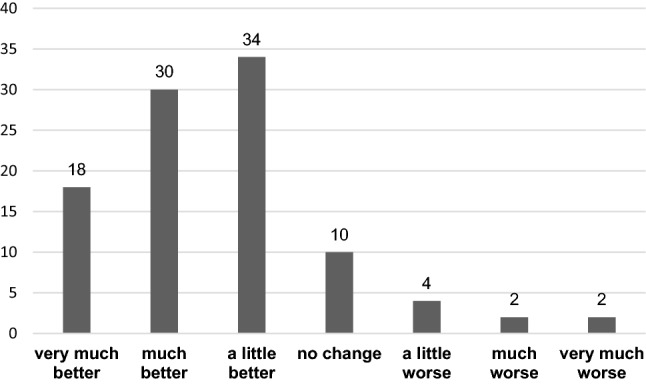
Patient global impression of improvement (PGI-I) six weeks post intervention*, *Values are percentages (%)

The primary feasibility outcome measure at 6 weeks was achieved by 34/50 (68%) of patients. Responses to IIEF-15 question 3 improved from 1.7 ± 1.6 to 3.2 ± 1.8 points (*p* < 0.05), while those for IIEF-15 question 4 improved from 1.3 ± 1.2 to 2.8 ± 1.7 points (*p* < 0.05). In addition, the IIEF-15 total score improved from 31.1 ± 15.1 at baseline to 50.0 ± 15.5 (*p* < 0.05). Based on the PGI-I questionnaire, a total of 41/50 (82%) patients felt better post intervention whereas 9/50 (18%) patients had no change or deterioration (Fig. 3). At six weeks follow-up 39/50 (78%) patients stated that they would undergo the procedure again.

Four out of six (66.7%) patients that had previously undergone endovascular arterial revascularization of erection-related arteries but had insufficient response with concomitant venous leak, reached the primary outcome measure. In these 4 patients, the IIEF-6 score increased from 4 ± 2.9 points pre intervention to 17.5 ± 6.4 post intervention.

## Discussion

Currently available therapeutic options for patients with venogenic ED, non-responsive to conservative measures, are either surgical or endovascular treatment. Various endovascular treatment options including embolization procedures for venous leak occlusion have recently been reported for treatment of patients with severe ED [6, 11, 21]. In the present study, venous embolization was performed via anterograde access via a deep dorsal penile vein using ultrasound guidance after intra-cavernosal injection of alprostadil. Subsequently, a NBCA-based liquid embolic agent mixed with ethiodol was used for safe and efficacious embolization as previously described [6]. Of note, anterograde access via a deep dorsal penile vein may be technically challenging due to a small venous caliber or spasm. On the other hand, retrograde catheterization of the internal and external pudendal veins via a transfemoral access may be even more cumbersome [13, 22].

Technical success rates of venous leak embolization were reported to be as high as 97%, which is in accordance with our study results (98%) [23]. In the present study, anterograde access was successful in all but one patient. This patient’s deep dorsal penile vein appeared hypoplastic, and it was not possible to advance a guide wire all the way to the radix penis to safely introduce a cannula. However, this patient eventually was treated successfully with secondary transcatheter embolization of his venous leak using transfemoral access.

Complication rates were reported to be as low as 5%, including mainly minor complications, whereas major complications such as pulmonary embolism were very rare (< 1%) [23]. In the present study, no patient experienced a major adverse event. However, asymptomatic pulmonary embolism was noted fluoroscopically in 2/50 (4%) patients.

The overall clinical success rate according to a meta-analysis is 59.8%, with a wide range from 22–100% including various embolization techniques with or without surgical cut down and both partial and full responses [6, 23–25]. In the present study, clinical success on 6-week follow-up was 68%. For full response, meaning sufficient erection to perform intercourse success rates in the literature rather tend to be within the lower range of the spectrum [21]. Follow-up for clinical success ranges between 1- and 30-months post embolization and durability of treatment was not available for all studies [23]. In the present study, 6 weeks follow-up was performed, which is rather on the shorter end of the range, but longer-term follow-up results are pending.

Several limitations of the present series need to be addressed. This all-comers prospective registry is limited by still a relatively small number of patients enrolled. Despite the prospective study design a control group is missing and a possible placebo effect cannot be fully eliminated. In addition, patients received tadalafil 5 mg once daily post venous embolization for six weeks. Of note, all patients included were non-responsive to maximum doses of PDE5i and 90% of patients were non-responders to intracavernous alprostadil at baseline. Thus, with the present analysis we may not be able to determine how the effect of the embolization procedure would have been without the use of PDE5i.

In conclusion, venous leak embolization via deep dorsal penile vein access using a liquid embolic agent was safe for all and efficacious in the majority of patients with severe venogenic ED on 6 weeks follow-up. Long-term follow-up results are pending and will be analyzed consecutively.

## Supplementary Information

Below is the link to the electronic supplementary material.

Supplementary file1 (DOCX 16 kb)
