# Supplementary material for: Venous Leak Embolization in Patients with Venogenic Erectile Dysfunction via Deep Dorsal Penile Vein Access: Safety and Early Efficacy
Source: Cardiovasc Intervent Radiol. 2023 Mar 22;46(5):610–6. doi: 10.1007/s00270-023-03412-2 (PMC10156837; doi:10.1007/s00270-023-03412-2)
Supplement: Supplementary file 1 — Supplementary file1 (DOCX 16 kb) [file 270_2023_3412_MOESM1_ESM.docx]

*Penile duplex sonography*

Duplex ultrasonography of the right and left cavernosal artery was performed using an Aplio 500 ultrasound device (Toshiba, Japan) 10 minutes after intracavernosal injection of 10 μg intracavernosal alprostadil (Caverject, Pfizer, Switzerland). The erectile response was graded visually from E0 to E5 as suggested by Broderick et al. (1). Grades E4 (full tumescence, medium rigidity) and E5 (full tumescence, full rigidity) are considered sufficient for penetration, whereas grades E0 to E3 are considered insufficient for penetration. At maximum erection, peak systolic velocity (PSV) and end diastolic velocity (EDV) were assessed. PSV of <30 cm/sec of right and left cavernosal arteries was considered as reduced arterial flow and justified the causal connection of ED to atherosclerosis-related arterial inflow obstruction (2). EDV of >5 cm/sec of right and left cavernosal arteries indicated venous leak (3).

*Computed tomography cavernosography*

For confirmation of venous leak diagnosis computed tomography cavernosography was performed post intra-cavernosal injection of 10 or 20 μg alprostadil. Ten minutes post injection a 23-G needle was inserted at the dorso-lateral side of the corpora cavernosum. Graduated injection of 10 to 20 cc of normal saline into corpora cavernosum at increasing flow rates starting at 0.1 ml/s was performed to determine the infusion velocity until penile tumescence of at least grade E4 was reached. Subsequently injection of 20-40 cc of 50% saline-diluted non-ionic iodinated contrast medium (350 mg ml− 1) with the above determined infusion velocity was performed. All injections were performed using a power injector. Computed tomography images were acquired using an Aquilion Prime scanner (Toshiba, Japan) with 80 × 0.5 mm collimation and 0.35 s gantry rotation time. Continuous scanning was performed under real time monitoring of venous contrast distribution extending from the upper brim of the true pelvis to the most distant level of the penis. Data constructive section thickness was 1 mm with a reconstruction increment of 1 mm for post-processing. For post-processing, multiplanar reformation using maximum intensity projection and volume rendering was applied.

**References**

1. Broderick GA, Arger P. Duplex Doppler ultrasonography: noninvasive assessment of penile anatomy and function. Semin Roentgenol. 1993;28(1):43-56.

2. Lee B, Sikka SC, Randrup ER, Villemarette P, Baum N, Hower JF, et al. Standardization of penile blood flow parameters in normal men using intracavernous prostaglandin E1 and visual sexual stimulation. J Urol. 1993;149(1):49-52.

3. Jung DC, Park SY, Lee JY. Penile Doppler ultrasonography revisited. Ultrasonography. 2018;37(1):16-24.
